# Effect of sense of control on emotional experience and response in Chinese undergraduate students

**DOI:** 10.3389/fpsyg.2025.1593500

**Published:** 2025-10-29

**Authors:** Li Yao, Yang Guo, Xu Zou, Yutong Zhang, Zhao Zhang, Suxuan Xing

**Affiliations:** School of Sports Training, Chengdu Sport University, Chengdu, China

**Keywords:** sense of control, lack of control, positive emotion, negative emotion, emotional response

## Abstract

**Objective:**

Sense of control significantly influences emotional well-being. A lack of control over stressors induces negative affect, while control buffers the impact of stress in experimental animals. However, it is also unclear whether control or lack of control alters emotional response to subsequent stimuli in humans. Therefore, the present study aimed to investigate the effects of sense of control on the emotional experience and response to emotional stimuli in undergraduate students.

**Methods:**

In Study 1, 488 participants were recruited to complete the questionnaires that included the Sense of Control Scale and Positive Affect and Negative Affect Scale (PANAS). In Study 2, 55 participants were randomly divided into a control group and a lack of control group. A concept identification task was used to manipulate the perceived control. The PANAS and a picture rating task were conducted before and after the manipulation.

**Results:**

In Study 1, The results revealed that the sense of control significantly predicted positive emotion (β = 0.28, *p* < 0.001) and negative emotion (β = −0.36, *p* < 0.001). In Study 2, compared to pre-test, the maintaining control group showed no significant changes in self-reported positive and negative emotion, nor in the valence ratings of emotion pictures after the manipulation. However, compared to pre-test, the lack of control group exhibited a decrease in self-reported positive emotion after the manipulation (*p* < 0.05), along with an increase in valence ratings for negative emotion pictures (*p* < 0.01). Additionally, the self-reported positive emotion in the lack of control group was lower than that in the control group after the manipulation (*p* < 0.05), while their valence ratings for negative emotion pictures were higher than those in the control group (*p* < 0.05). That is, maintaining control did not change the variables, while lack of control was associated with a decrease in positive affect and with valuing negative images as less negative.

**Conclusion:**

Control did not significantly alter the emotional experience or response of individuals to emotional stimuli, whereas lack of control led to a decrease in positive affect and a decreased response to negative stimuli at the behavioral level.

## 1 Introduction

Organisms are adaptive and emotions arise in response to a stimulus when it is appraised as meaningful for a currently active goal, as well as varying in intensity (strong or weak) and valence (positive or negative) (Lavi et al., 2019). However, the same stimuli can produce different emotional responses across individuals including subjective experiential, physiological, and behavioral responses. Some studies have found that depressed individuals displayed blunted emotional reactivity to both pleasant and unpleasant stimuli compared with healthy individuals (Bylsma et al., 2008; Rottenberg et al., 2005). In contrast, study also have discovered that depressed individuals exhibited attenuated emotional reactivity to pleasant stimuli but potentiated emotional reactivity to unpleasant stimuli (Wu et al., 2017). Although these findings have been inconsistent, they indicate that depressed individuals show emotional impairments (Sloan and Sandt, 2010; Vanderlind et al., 2020).

Lack of control is one of the critical factors that induces individuals to develop mood disorders such as depression (Maier and Seligman, 2016). Previous studies in experimental animals have demonstrated that differences in perceived control lead to variations in emotional responses (Maier and Seligman, 2016; Yao et al., 2019). Control over stressors leads individuals to exhibit appropriate emotional reactivity to negative and positive stimuli. That is, individuals are unpleasant to negative stimuli and pleasant to positive stimuli. However, lack of control over stressors in mice results in an increased negative emotional experience, but a decreased emotional reactivity to subsequent positive stimuli (Yao et al., 2021). It is well known that experimental animals live in laboratories with a homogeneous environment, in contrast to the complexity and variety of human life. Therefore, when control/lack of control is manipulated, do human participants exhibit different emotional responses to emotional stimuli than do those of experimental animals?

Previous studies have demonstrated that perceived control has protective effects and affective valuation (Wang and Delgado, 2021; Wang et al., 2021). It has been found that older adults who received control-relevant interventions over daily events showed a greater health (Rodin and Langer, 1977), and the perceptions of control buffered daily stress when adults reported strong control on days (Dan et al., 2024; Diehl and Hay, 2010). Moreover, internal control beliefs are sufficient to increase subjective positive feelings in adults (Stolz et al., 2020). These results indicate that a strong sense of control is associated with higher positive emotion and lower negative emotion (Diehl and Hay, 2010). Studies on individuals who experienced childhood trauma have indicated that sense of control altered their sensitivities to daily emotional events (Infurna et al., 2015). Individuals with childhood trauma who reported higher levels of sense of control exhibited stronger increases in well-being when experiencing positive events and stronger decreases in well-being with negative events. Therefore, if sense of control is experimentally manipulated, do control/lack of control elicit different emotional experience, or alter the responses to subsequent emotional stimuli in healthy young adults?

Therefore, the present study employed a dual design, consisting of two studies. Study 1 investigated the relationship between sense of control and emotion, and Study 2 explored the effect of sense of control on emotional experience and emotional response among Chinese undergraduate students. In Study 1, sense of control was assessed by the sense of control scale, and the subjective emotion was assessed by the positive affect and negative affect scale (PANAS). In Study 2, a concept identification task was used to manipulate sense of control. Image rating task, requiring participants to rate pleasant, neutral and unpleasant pictures for valence and arousal, was used to assess behavioral response to emotional stimuli. The image rating task and the PANAS were administered before and after the manipulation. We predicted that maintaining control would result in an increase in positive affect and a decrease in negative affect, but would have no impact on the valence or arousal ratings of subsequent positive and negative emotional pictures. Conversely, loss of control might lead to a decrease in positive affect and an increase in negative affect. Specifically concerning picture ratings, loss of control would lower valence ratings for positive emotion pictures while increasing the valence ratings for negative emotion pictures, though this condition would not affect the arousal ratings for either subsequent positive or negative emotion pictures.

## 2 Materials and methods

### 2.1 Participants

Participants were 500 healthy undergraduates in Study 1 and 56 healthy undergraduates in Study 2. All participants were from the Chengdu Sport University. In Study 1, the criteria for the participant recruited included: (1) Voluntary, (2) no self-reported history of mental disorders, and (3) normal or rectified to normal vision. 12 participants who did not respond to all the items were excluded, and thus 488 (374 man, 114 woman, M_*age*_ = 19.47 ± 0.99) responses were ultimately used. In Study 2, the sample size for the present study was calculated using the G*power 3.1 program (Jung et al., 2024). The analysis revealed that minimum sample size was calculated to be 54 for a design with repeated-measure analysis of variance (ANOVA), an alpha error probability of 0.05 (two-tailed), a power of 0.95, and a medium effect size (0.25). the criteria for the participant recruited included: (1) no self-reported history of mental disorders, (2) normal or rectified to normal vision, and (3) no color blindness. A researcher blinded to participants’ identities and not involved in enrollment or testing used a random number table to allocate participants to control group and lack of control group, but one participant in the control group who withdrew from the experiment was excluded (control group: *n* = 27, M_*age*_ = 20.07 ± 0.21; lack of control group: *n* = 28, M_*age*_ = 19.86 ± 0.18).

### 2.2 Measures

#### 2.2.1 Sense of control scale

The 12-item sense of control scale includes two subscales, personal mastery and perceived constraints, to assess how much they generally felt in control of their lives (Lachman and Weaver, 1998). On each item, subjects were asked to evaluate the degrees of agreement or disagreement with each statement on a seven-point Likert scale (1 = strongly disagree, 7 = strongly agree). The perceived constraints scale was reverse scored, and both scales were summed with higher scores indicating a greater sense of control. The Chinese version of sense of control scale (Yu et al., 2018) was used in the current study with the Cronbach’α = 0.78 (personal mastery) and 0.82 (perceived constraints).

#### 2.2.2 Positive affect and negative affect scale (PANAS)

PANAS was used to assess immediate emotional states, including the 10-item positive affect (PA) and negative affect (NA) scales (Watson et al., 1988). The present study used the Chinese revision of PANAS by Qiu et al. (2008). The PANAS instructions required participants to rate their current state based on the descriptions provided. The scale is scored on a 5-point scale (1 = almost none to 5 = extremely much), and the average score of the PA and NA subscales is calculated. The Cronbach’s alpha of the PA and NA subscales was 0.92 and 0.93, respectively.

### 2.3 Concept identification task

**FIGURE 1:**
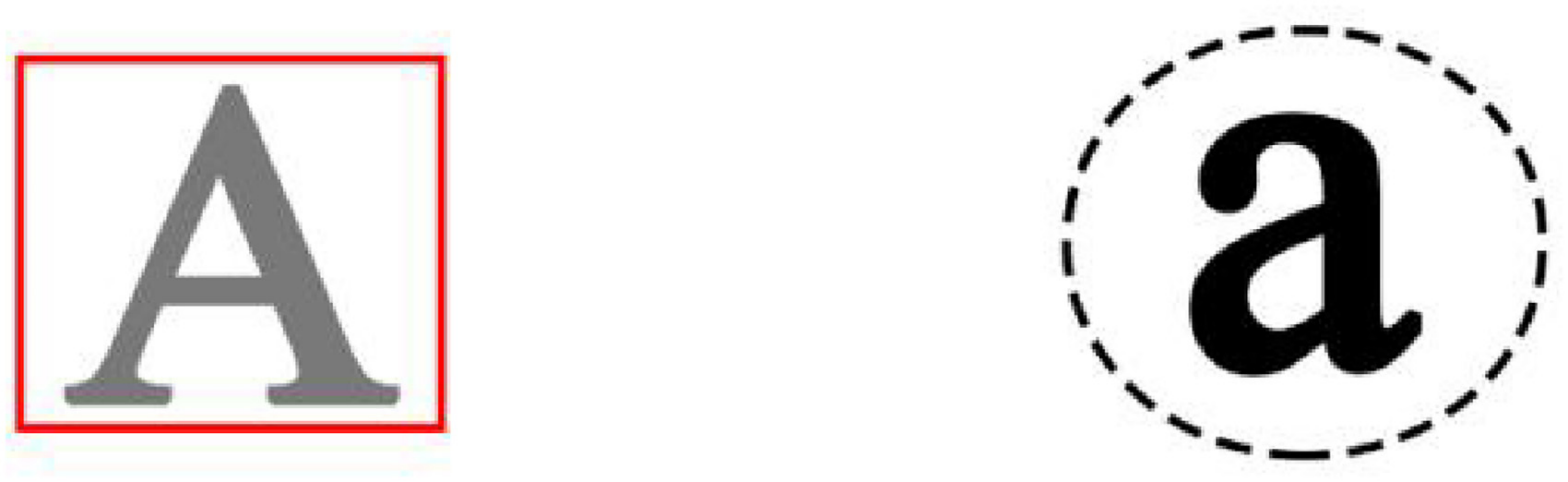
Example of concept identification task.

The concept identification task was created by Pittman and Pittman (1979), and used by Whitson and Galinsky (2008) to manipulate the sense of control. Participants were presented with ten pairs of symbols in each block, and each pair was presented for no more than 15 s. These symbols varied in five dimensions: letters size (uppercase or lowercase), letter color (gray or black), border color surrounding the letters (red or black), border line surrounding the letters (solid or dashed), border shape surrounding the letters (circle or square) (Figure 1). Each block pseudo-randomly selected one attribute (e.g., circle) as a preset correct concept. Participants were required to learn the concept from the following feedback. Participants were instructed to determine which side of screen displayed the correct concept and respond by pressing a key on the keyboard. If participants thought the preset correct concept was on the left, they pressed “←” key; if they thought the preset correct concept was on the right and pressed “→” key, followed by a correct or incorrect feedback. In each block, the response in the first trial was entirely based on one’s own guess, and the subsequent response in next nine trials were required to choose correctly as often as possible according to the previous feedback. The practice experiment with one block was performed to get used to the task. After the practice experiment, participants completed another four blocks in the formal experiment. For the control group, participants received completely correct feedback. For the lack of control group, participants received feedback with a 50% probability of being correct and a 50% probability of being incorrect.

### 2.4 Image rating task

Picture stimuli (size: 1024 × 768, 72 pixels per inch) included 20 pleasant, 20 neutral, and 20 unpleasant pictures from the International Affective Picture System (IAPS) (Lang et al., 1999; Pollatos et al., 2005). In order to examine the effectiveness of picture stimuli, *a priori* experiment recruited twenty-four participants to rate the pictures with regard to valence (1 = very unpleasant to 9 = very pleasant) and arousal (1 = very weak to 9 = very strong) on 9-point scales. Pleasant, neutral and unpleasant pictures differed significantly in the ratings of valence [*F*_(2_, _46)_ = 238.03, *p* < 0.001, $\eta_{\text{p}}^{2}$ = 0.93]. Pleasant pictures (7.31 ± 0.88) were significantly higher valence rating than both neutral (5.00 ± 0.79, *p* < 0.001, 95% CI_*diff*_: 1.82 to 2.80) and unpleasant (2.06 ± 0.71, *p* < 0.001, 95% CI_*diff*_: 4.48 to 6.01) pictures, and neutral pictures were significantly higher valence rating than unpleasant pictures (*p* < 0.001, 95% CI_*diff*_: 2.36 to 3.51). Additionally, arousal ratings among pleasant, neutral and unpleasant pictures also differed significantly [*F*_(2_, _46)_ = 46.69, *p* < 0.001, $\eta_{\text{p}}^{2}$ = 0.67]. Arousal ratings were higher for both pleasant and unpleasant pictures than for neutral [pleasant vs. neutral: (6.76 ± 1.03) vs. (4.39 ± 1.22), *p* < 0.001, 95% CI_*diff*_: 1.75 to 3.00; unpleasant vs. neutral: (6.92 ± 1.38) vs. (4.39 ± 1.22), *p* < 0.001, 95% CI_*diff*_: 1.59 to 3.59) pictures, indicating the effectiveness of selected emotional picture stimuli. In picture rating task, a fixation point was first presented in the center of the screen for 1 s, and a blank screen was presented for 500 ms. After that, a picture was randomly presented for 6 s, followed by the valence and arousal ratings on the 9-point scales, respectively. The inter-stimulus-interval (ISI) following a random blank screen was 1–5 s before the next trial was presented. The total time of the task lasted about 10 min.

### 2.5 Procedure

In Study 1, participants were instructed to complete the questionnaires including the sense of control scale and PANAS. In Study 2, participants were firstly introduced to the experiment information. Next, participants completed the PANAS, and then they performed the image rating task in pre-test. Then, the concept identification task was used to manipulate the sense of control, and participants were also required to rate the degree of perceived control on a 7-point scale (1 = almost none to 7 = very much). Finally, the PANAS and the image rating task were performed again.

After the experiment, all participants were debriefed and asked about the true purpose of the study, and all their questions were truthfully answered. All participants failed to correctly state the study’s actual intent.

### 2.6 Statistical analysis

Data analysis was performed using SPSS version 22.0. In Study 1, Pearson correlation analysis and regression analysis were performed to examine the relationship between sense of control and emotions. In Study 2, to assess the effectiveness of perceived control manipulation, an independent samples *t*-test was conducted to determine differences in the rating of perceived control between the control and lack of control groups. A 2 (group: control group vs. lack of control group) × 2 (time: pre-test vs. post-test) repeated measures ANOVA was conducted on the scores of PANAS, valence and arousal ratings of different emotional pictures, respectively. *Post hoc* comparisons were corrected with the Bonferroni correction. The significance level adopted was 5% (*p* < 0.05), with 95% confidence intervals (CI). The 95% confidence intervals for difference (CI_*diff*_) measure was computed between conditions.

## 3 Results

### 3.1 Common method bias test

Harman’s single-factor test was conducted to assess the potential common method bias. Four factors with eigenvalues greater than 1 were extracted, with the largest factor accounting for only 24.69% of the variance, which is well below the threshold of 40%. These results indicate that the common method bias did not significantly affect the study results.

### 3.2 Correlations between sense of control and emotion in study 1

In Study 1, the results of the correlation analysis showed a significant positive relationship between sense of control and positive emotion (*r* = 0.28, *p* < 0.001), but a significant negative relationship between sense of control and negative emotion (*r* = −0.36, *p* < 0.001). The results of the regression analysis indicated that the sense of control significantly predicted positive emotion (β = 0.28, *p* < 0.001, 95% CI: 0.17 to 0.33) and negative emotion (β = −0.36, *p* < 0.001, 95% CI: −0.39 to −0.25).

### 3.3 Effects of perceived control on emotion in study 2

#### 3.3.1 The sense of control intervention manipulation

In Study 2, in order to examine the effectiveness of perceived control intervention manipulation, an independent samples *t*-test revealed that the rating of perceived control was higher in the control group than that in the lack of control group [(5.7 ± 0.8) vs. (3.6 ± 0.8), *t*_53_ = 9.18, *p* < 0.001], indicating that the concept identification task was successful in manipulating the perceived control.

#### 3.3.2 Effect of perceived control on emotion experience

**FIGURE 2:**
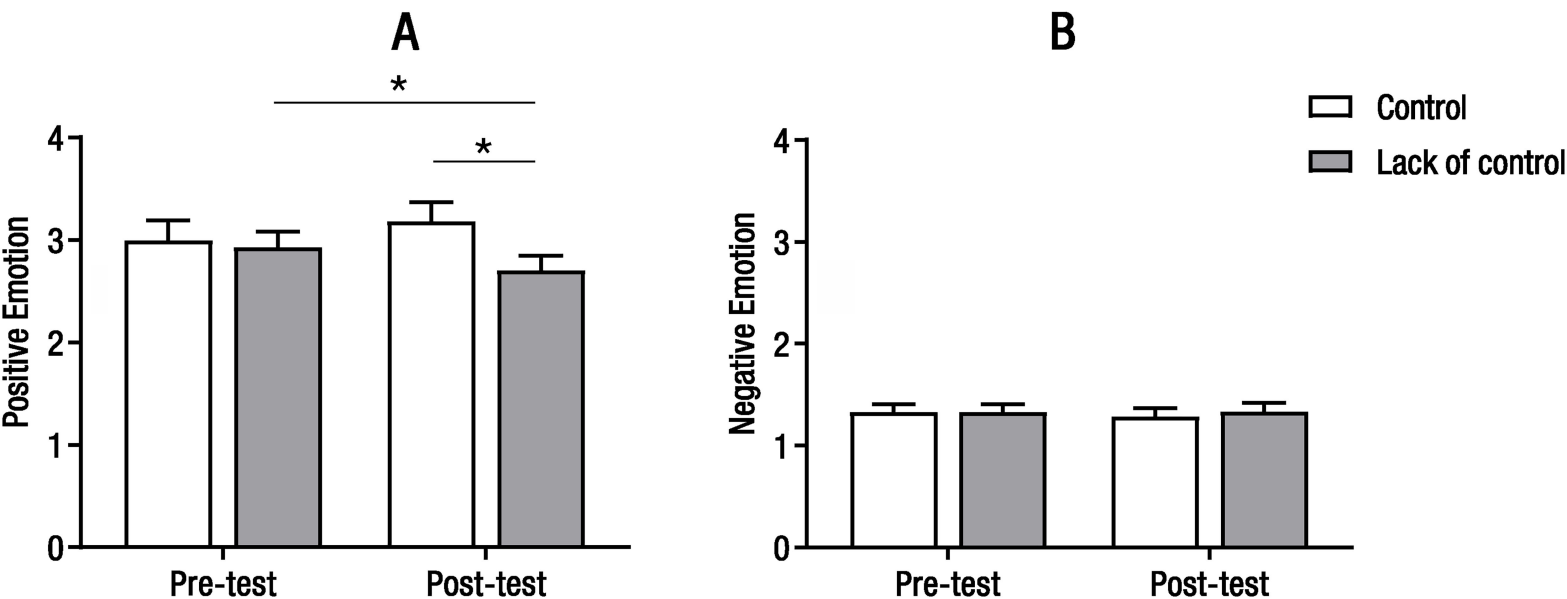
Results of self-reported positive emotion **(A)** and negative emotion **(B)** in the control and the lack of control groups (*p < 0.05).

In order to examine the emotion changes after perceived control intervention, the repeated measures ANOVA for self-reported positive emotion showed that there were no significant main effects of group [*F*_(1_, _53)_ = 1.44, *p* = 0.235] or time [*F*_(1_, _53)_ = 0.10, *p* = 0.754], but a significant interaction between group and time [*F*_(1_, _53)_ = 9.03, *p* = 0.004, $\eta_{\text{p}}^{2}$ = 0.15, Figure 2A and Table 1] were observed. Follow-up analyses revealed that there was no significant difference between pre-test and post-test for the control group [(3.00 ± 1.00) vs. (3.19 ± 0.97), *p* = 0.065], but positive affect scores were significantly lower in the post-test than those in the pre-test for the lack of control group [(2.70 ± 0.77) vs. (2.93 ± 0.79), *p* = 0.021, 95% CI_*diff*_: 0.35 to 0.42]. In addition, there was no significant difference between the control and lack of control groups at the pre-test (*p* = 0.78), whereas positive affect scores were significantly lower in the lack of control group than in the control group at the post-test (*p* = 0.047, 95% CI_*diff*_: 0.01 to 0.96). The repeated measures ANOVA for self-reported negative affect showed no significant main effects of group, or time, or the interaction between group and time [*F*(1, 53) = 0.052, 0.19, 0.35, *p* = 0.821, 0.663, 0.557, Figure 2B]. These results suggest that control did not significantly alter individuals’ self-reported positive and negative emotions, whereas lack of control resulted in a decrease in self-reported positive emotion.

**TABLE 1:** Descriptive statistics of PA and NA scale scores (pre-test and post-test) for control and lack of control groups.

Group	Time	PA scores (M ± SD)	NA scores (M ± SD)
Control	Pre-test	3.00 ± 1.00	1.33 ± 0.36
	Post-test	3.19 ± 0.97	1.28 ± 0.43
Lack of control	Pre-test	2.93 ± 0.79	1.33 ± 0.41
	Post-test	2.70 ± 0.77	1.34 ± 0.43

#### 3.3.3 Effect of perceived control on emotional response

**FIGURE 3:**
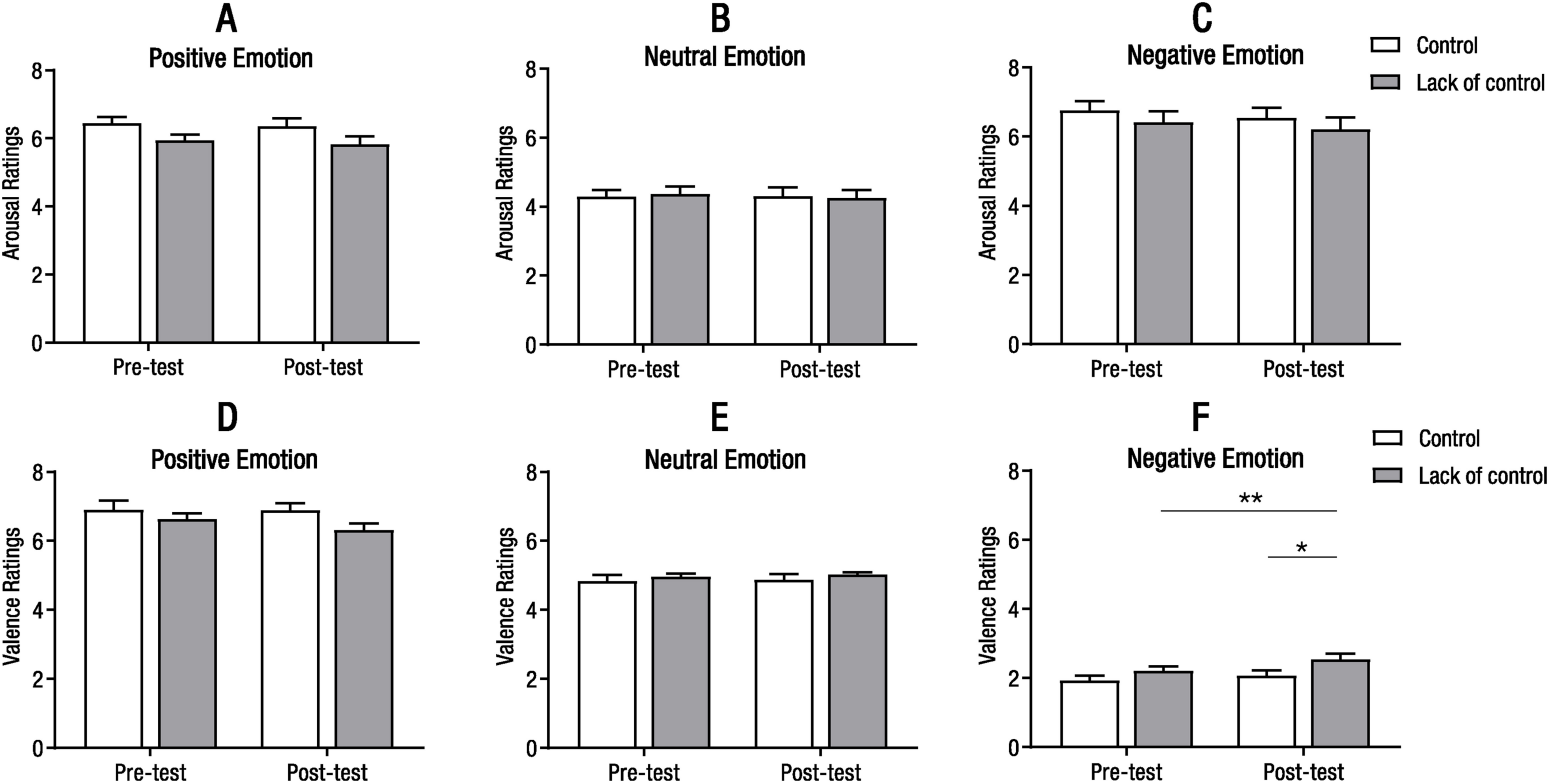
Results of arousal **(A–C)** and valence **(D–F)** ratings of different emotion pictures in the control and the lack of control group (*p < 0.05, **p < 0.01).

For arousal ratings to positive, neutral, and negative pictures, repeated measures ANOVA showed that there were no significant main effects of group, or time, or the interaction between group and time [positive pictures: *F*(1, 53) = 3.54, 1.22, 0.05, *p* = 0.065, 0.274, 0.82, Figure 3A; neutral pictures: *F*(1, 53) = 0.001, 0.41, 0.72, *p* = 0.982, 0.524, 0.4, Figure 3B; negative pictures: *F*(1, 53) = 0.68, 3.53, 0.004, *p* = 0.415, 0.066, 0.947, Figure 3C], indicating that arousal ratings to different emotional pictures were not significantly different in the control and lack of control groups. Then, a repeated-measures ANOVA showed that there were no main effects of group, or time, or intervention between group and time for valence ratings to positive and neutral pictures [positive pictures: *F*(1, 53) = 2.55, 1.61, 1.29, *p* = 0.117, 0.211, and 0.26, Figure 3D; neutral pictures: *F*(1, 53) = 0.61, 0.66, 0.09, *p* = 0.437, 0.421, 0.764, Figure 3E], whereas the main effect of time for valence ratings of negative pictures was significant [*F*(1, 53) = 7.94, *p* = 0.007, $\eta_{\text{p}}^{2}$ = 0.13, Figure 3F and Table 2]. Follow-up analyses showed that the valence ratings were significantly higher in the post-test than in the pre-test for the lack of control group [(2.55 ± 0.85) vs. (2.22 ± 0.68), *p* = 0.005, 95% CI_diff_: 0.10 to 0.57], whereas there was no significant difference between pre-test and post-test for the control group [(1.94 ± 0.74) vs. (2.07 ± 0.83), *p* = 0.275]. In addition, there was no significant difference between the control and lack of control groups in the pre-test (*p* = 0.151), but in the post-test, the valence ratings to negative pictures were significantly higher in the lack of control group than in the control group (*p* = 0.038, 95% CI_diff_: 0.03 to 0.94). These results showed that control over the task did not change individuals’ ratings of pleasantness and arousal to emotional pictures, and lack of control also did not change their arousal rating to emotional pictures. However, lack of control increased individuals’ ratings of the pleasantness in response to negative pictures, suggesting that control or lack of control did not change the intensity of the internal responses elicited by emotional pictures, whereas lack of control attenuated individuals’ responses to negative stimuli.

**TABLE 2:** Descriptive statistics of valence and arousal ratings of different emotional pictures (pre-test and post-test) for control and lack of control groups.

Group	Time	Valence ratings (M ± SD)			Arousal ratings (M ± SD)		
		Positive	Neutral	Negative	Positive	Neutral	Negative
Control	Pre-test	6.91 ± 1.36	4.84 ± 0.93	1.94 ± 0.74	6.44 ± 0.96	4.29 ± 1.00	6.76 ± 1.38
	Post-test	6.89 ± 1.08	4.87 ± 0.93	2.07 ± 0.83	6.36 ± 1.15	4.31 ± 1.33	6.55 ± 1.51
Lack of control	Pre-test	6.63 ± 0.87	4.97 ± 0.47	2.22 ± 0.68	5.95 ± 0.90	4.37 ± 1.14	6.41 ± 1.67
	Post-test	6.32 ± 1.02	5.03 ± 0.36	2.55 ± 0.85	5.83 ± 1.22	4.25 ± 1.25	6.21 ± 1.77

## 4 Discussion

In Study 1, there is a positive correlation between the sense of control and positive emotions, but a negative correlation with negative emotions. Moreover, the sense of control can, to some extent, predict an individual’s emotional state. People with high levels of control have high levels of positive emotion and low levels of negative emotion. Previous studies have shown that the sense of personal control predicts psychological distress (Ross and Mirowsky, 2013) and subjective well-being. The sense of being in control of the outcomes in one’s personal life can decrease distress. Of course, individuals with a strong sense of control have more positive emotions, and fewer negative emotions. However, low levels of personal control significantly increase depression and anxiety (Ross and Mirowsky, 2009). These results consistently address the correlation between sense of control and emotion. Therefore, Study 2 was used to examine the effect of perceived control on emotion.

In Study 2, to assess whether the sense of control influenced participant ratings of emotional experience and responding, the concept identification task was used to manipulate the sense of control (Whitson and Galinsky, 2008; Yao et al., 2022). Furthermore, participants were instructed to make emotion ratings for their feelings and emotion images before and after the sense of control manipulation. The results indicated that participants who maintained their sense of control over the task did not exhibit significant changes in self-reported positive or negative emotions. In contrast, participants in the lack of control group exhibited a significant decrease in self-reported positive emotions after the manipulation with lack of control over the task. These findings demonstrated that lack of control has deleterious effects. Previous study with laboratory animals has shown that a lack or loss of control over stressors leads to increased negative emotional and behavioral responses to aversive stimuli, and reduced seeking behavior for rewarding stimuli, whereas control results in decreased negative emotional responses to aversive stimuli (Maier and Seligman, 2016; Yao et al., 2019). However, in Study 2, participants with control over the task did not exhibit significant changes in positive or negative emotions. A possible explanation is that stressors *per se* have deleterious effects in previous studies, and that these effects would be blocked when control was added, but in our study the concept identification task used to manipulate the sense of control did not involve emotionally relevant stimulus materials, which may have resulted in no observable positive effect after gaining control over the task. Unfortunately, previous studies with laboratory animals did not completely separate factors of aversive stimuli from control, which may have influenced each other. Aversive stimuli *per se* can increase their negative emotions, and then these negative emotions are reduced following perceived control over aversive stimuli (Maier and Seligman, 2016; Yao et al., 2019). Additionally, the results of this study may also suggest that participants with undergraduates, which have rich experiences of control in life, do not exhibit significant changes in emotional experiences from brief control over an emotionally irrelevant task, whereas the lack of control over the task indeed has deleterious effects.

According to classic literature of learned helplessness, control and lack of control were manipulated by establishing the conditional probability of an outcome following a response (or the absence of that response) (Maier and Seligman, 1976). In the present study, the concept identification task employed a similar principle by establishing differential feedback contingencies in control and lack of control groups to manipulate perceived control (Whitson and Galinsky, 2008). In the control group, participants acquired the correct concept and developed control over the task based on 100% correct feedback. In contrast, the lack of control group only received 50% correct feedback, preventing them from acquiring the correct concept and thus lacking control over the task. The manipulation check indicated group differences for sense of control, but these findings did not exclude the possibility that motivation, emotion, or engagement were confounding factors. Therefore, the low engagement of participants in the control group might also be the reason why their positive and negative emotions did not show significant changes.

Does this sense of control over the task affect an individual’s emotional response to new stimuli? In life, people experience both good and bad events, and their emotional responses to these events may influence their behavioral performance. Therefore, Study 2 was used to assess individuals’ emotional responses to external emotion stimuli after control or lack of control over the task. The results showed that there were no significant changes in the arousal or valence ratings for positive, neutral, or negative emotional pictures following control over the task, and there were also no significant changes in the arousal ratings of different emotional pictures following lack of control over the task, but the valence ratings of negative emotional pictures significantly increased. This result indicated that control over the task did not induce individuals’ changes in response to emotional stimuli, whereas lack of control led to a decreased response at the behavioral level, specifically to negative stimuli rather than positive stimuli. A possible explanation for this result is that control has a protective effect (Wang and Delgado, 2021), and healthy individuals exhibit appropriate emotional responses when emotional stimuli were presented following control over the task; whereas lack of control with deleterious effects leads to a decrease in response to negative stimuli, and this decrease alleviates the individual’s negative emotions when they are faced with negative stimuli. In previous studies, individuals with lack or loss of control, which was one of the methods to develop emotional disorders such as depression, exhibited fewer escape and resistance behaviors for negative events (Dan et al., 2024; Yao et al., 2019), and this change of behavior may be explained by a decrease in response to negative stimuli.

Disturbance of mood is one of the most salient features of depression. According to the Emotional Context Insensitivity (ECI) theory (Rottenberg et al., 2005; Sun et al., 2022), depressed individuals exhibit insensitivity to emotional stimuli and changes in emotional reactivity. In Study 2, individuals who lacked a sense of control over the task reported less positive emotional experience and a less negative emotional response to negative images. These results indicated that lack of control produced maladaptive emotional reactivity, which may be important in the development of depression and perhaps also one of the trans-diagnostic features of depression. Therefore, in our life, it is necessary to avoid depriving individuals’ sense of control, and reduce the risk of emotional disorders.

In conclusion, control over the task did not produce significant changes in self-reported emotional experience or response to emotional stimuli. However, lack of control over the task had deleterious effects, but these effects were asymmetrical. Specifically, lack of control led to a decrease in positive affect and a decreased response to negative emotional stimuli at the behavioral level. Therefore, a sense of control is an important factor to maintain mental health.

However, there are some limitations. First, self-reports and behavioral responses were used to assess emotional experience and emotional responses in this study, and although they were an effective way to measure changes in emotions of individuals, they did not measure physiological responses (such as blink reflex and skin conductance). Second, in the image rating task, the same images were used in pre-test and post-test. Although the use of between-subjects design and randomization of images, habituation may still occur. Future research should employ parallel forms of the assessment for pre-test and post-test designs. Third, the sense of control was manipulated by a conceptual judgment task, and the task did not involve a threatening or emotional component. Therefore, future research could design emotionally relevant tasks to manipulate individuals’ sense of control, exploring the impact of control or lack of control on emotions and behaviors in the context of threat and emotion. Fourth, the study sample consisted of undergraduates with a limited number of participants, which limited the generalizability of the findings. While previous research has indicated a positive correlation between sense of control and well-being in middle-aged and older adults (Toh et al., 2020), future research is encouraged to explore whether interventions targeting sense of control can further enhance well-being in this population.

Our findings have significant implications for individual mental health. The need of control is a biological imperative for survival and essential for an individual’s wellbeing (Leotti et al., 2010). Sustaining and strengthening control is a long-term goal worth pursuing.

## Data Availability

The original contributions presented in this study are included in this article/supplementary material, further inquiries can be directed to the corresponding author.
